# Intestinal manipulation affects mucosal antimicrobial defense in a mouse model of postoperative ileus

**DOI:** 10.1371/journal.pone.0195516

**Published:** 2018-04-13

**Authors:** Kathy Stein, Lena Hieggelke, Bianca Schneiker, Mariola Lysson, Burkhard Stoffels, Sabine Nuding, Jan Wehkamp, Judith Kikhney, Annette Moter, Joerg C. Kalff, Sven Wehner

**Affiliations:** 1 Department of Surgery, University Hospital of Bonn, Bonn, Germany; 2 Dr. Margarete Fischer-Bosch-Institute for Clinical Pharmacology, Stuttgart, Germany; 3 Internal Medicine I, University Hospital of Tübingen, Tübingen, Germany; 4 Institute of Microbiology and Hygiene/Biofilmcenter, Charité-University Medicine, Berlin, Germany; "INSERM", FRANCE

## Abstract

**Aim:**

To explore the effects of abdominal surgery and interleukin-1 signaling on antimicrobial defense in a model of postoperative ileus.

**Methods:**

C57BL/6 and Interleukin-1 receptor type I (IL-1R1) deficient mice underwent intestinal manipulation to induce POI. Expression of mucosal IL-1α, IL-1β and IL-1R1 and several antimicrobial peptides and enzymes were measured by quantitative PCR or ELISA, western blotting or immunohistochemistry. Bacterial overgrowth was determined by fluorescent in-situ hybridization and counting of jejunal luminal bacteria. Translocation of aerobic and anaerobic bacteria into the intestinal wall, mesenteric lymph nodes, liver and spleen was determined by counting bacterial colonies on agar plates 48h after plating of tissue homogenates. Antimicrobial activity against *E*. *coli* and *B*. *vulgatus* was analyzed in total and cationic fractions of small bowel mucosal tissue homogenates by a flow cytometry-based bacterial depolarization assay.

**Results:**

Jejunal bacterial overgrowth was detected 24h after surgery. At the same time point, but not in the early phase 3h after surgery, bacterial translocation into the liver and mesenteric lymph nodes was observed. Increased antimicrobial activity against *E*. *coli* was induced within early phase of POI. Basal antimicrobial peptide and enzyme gene expression was higher in the ileal compared to the jejunal mucosa. The expression of lysozyme 1, cryptdin 1, cryptdin 4 and mucin 2 were reduced 24h after surgery in the ileal mucosa and mucin 2 was also reduced in the jejunum. Postoperative IL-1α and IL-1β were increased in the postoperative mucosa. Deficiency of IL-1R1 affected the expression of antimicrobial peptides during homeostasis and POI.

**Conclusion:**

Small bowel antimicrobial capacity is disturbed during POI which is accompanied by bacterial overgrowth and translocation. IL-1R1 is partially involved in the gene expression of mucosal antimicrobial peptides. Altered small bowel antimicrobial activity may contribute also to POI development and manifestation in patients undergoing abdominal surgery.

## Introduction

Post-operative ileus (POI) is an iatrogenic impairment of propulsive gastrointestinal motility, frequently occurring after abdominal surgery. Although substantial efforts have been made in POI research, an effective clinical therapy does not exist and current therapeutic options are restricted to supportive measures [1, 2]. Response The pathogenesis of POI depends on neurogenic and inflammatory mechanisms that mainly remain limited to the muscularis externa of the GI tract [3, 4]. The impaired postoperative motility leads to passage disturbances which is discussed to results in bacterial overgrowth and some studies detect impaired mucosal barrier integrity after surgery [5, 6]. In consequence, translocation of microorganisms [7] from the intestinal lumen into the sterile bowel wall or distant organs aggravates the risk of inflammation which further compromises the intestinal barrier. This vicious cycle of increasing inflammation and decreasing barrier integrity can result in systemic infection and multiple organ dysfunctions [8].

During POI, the regular gastrointestinal peristalsis, as the major factor preventing luminal bacterial overgrowth of the small bowel, is disturbed. Therefore, additional antimicrobial mechanism may be of important relevance to protect the epithelium from an increasing microbial challenge. Such mechanisms include the secretion of antimicrobial peptides (AMPs) and production of a tight layer of mucus that both help to defend luminal pathogens and reduce the overall bacterial burden at the epithelial surface [9]. AMPs are polypeptides that have the ability to kill bacteria by interfering with membrane proteins [10]. They are produced by intestinal epithelial cells (IECs) that arise from the stem cells located at the base of the crypts [11] or by infiltrating leukocytes (i.e. human alpha defensins (HD) 1–4, lysozyme C type M) [12]. Some are constitutively expressed (i.e. HD 5–6, referring to the class of cryptdins in mice), while others are induced during inflammation (β-defensins, lysozyme C type P, C-type lectins like REGIIIα/γ) [12]. The secretion of IEC-derived AMPs is enhanced by or in some cases even dependents on the presence of commensal bacteria [12–14].

The molecular regulation of intestinal AMP production is part of several ongoing studies. In the lung and skin, two organs that also are continuously challenged by environmental microorganisms, interkeukin-1(IL-1)α and IL-1β, which both target the IL-1R type I (IL-1R1) [15], have been shown to signal AMP production [16–19]. In the bowel IECs serve as sensor cells in recognition of microbial products by expressing TLR receptors and activate the innate immune system via the adaptor molecule MyD88 [13]. In chronic inflammatory bowel diseases (IBD), the antimicrobial barrier is thought to be a central pathophysiological component [20] and increased NF-κB activation, associated with increased IL-1β expression in macrophages and IECs was shown to correlate with disease activity [21, 22]. However, although IL-1R1 is known to be expressed in the intestinal mucosa by a variety of different cell types [23, 24] it is unknown if IL-1R1 signaling affects intestinal AMP regulation by IECs during acute intestinal inflammation i.e. during POI development. Herein we hypothesized that the intestinal antimicrobial defense is disturbed during POI and that the IL-1R1 may contributes to the regulation of the mucosal antimicrobial response.

## Materials and methods

### Ethics statement

All experiments were approved by the department #84 of the "Ministry for Environment, Agriculture, Conservation and Consumer Protection of the State of North Rhine-Westphalia" (Landesamt für Natur, Umwelt und Verbraucherschutz, LANUV)

### Animals

C57BL/6 (WT) mice (Janvier, France) and Il1r^tm1Imx^ (IL-1R1 deficient) mice from Jackson Laboratories (Charles River, Germany) were kept under specific pathogen-free conditions in the animal housing facility of the University Hospital of Bonn (Germany). All experiments were performed in 8–12 week-old male mice with an average body weight of 20 to 25g.

### Animal model of POI

POI was induced by standardized small bowel manipulation as described previously [25]. In brief, after laparotomy and eventration of the intestine, the small bowel is rolled twice between two moistened cotton sticks. Afterwards the intestine is laid back into the abdominal cavity and the abdomen closed by a two-layer suture. Non-operated mice were taken as controls in these experiments.

### Fluorescence in situ hybridization (FISH)

For FISH jejunum was sampled, maintaining the gut content in place as described before [26]. The tissue was fixed immediately in PBS containing 50% (v/v) ethanol (pH 7.4) and 3.7% (v/v) formaldehyde at 4°C, embedded in polymerizing resin (Technovit 8100, Kulzer), and sectioned as described before [26, 27].

FISH experiments were performed as published [28]. Briefly, 2μm sections were enzymatically permeabilized and hybridized with Cy3-labelled probe EUB338 [29], complementary to the 16S rRNA in most microorganisms, and combined with Cy5-labelled FISH probe NON-EUB338 to exclude unspecific probe binding [30]. Furthermore, the nucleic acids were stained with DAPI. After incubation in a dark, humid chamber for 2 hours at 50°C, the slides were rinsed with sterile water, dried, and mounted using Vectashield mounting medium (Vector Laboratories, Burlingame, CA). Image analysis was done using an epifluorescence microscope (Axioplan 2; Carl Zeiss, Germany) equipped with narrow band filter sets (AHF Analysentechnik, Germany). Image acquisition was performed with an AxioCam MRm (Zeiss, Germany) making use of the AxioVision 4.4 software.

For quantification of bacterial load in the gut lumen, the area of EUB338 FISH-signal was quantified in twelve microscopic fields per animal per group using the digital image analysis software Digital Image Analysis in Microbial Ecology (DAIME) [31]. EUB338- positive areas that were also NON-EUB338-positive were excluded from the quantification.

### Bacterial translocation assay

Intestinal manipulation (IM) was performed under sterile conditions. Mesenteric lymph nodes (MLN), liver, lung and spleen from unmanipulated (CTL) or IM (IM3h, IM24h) mice were removed and organs transferred into sterile medium containing 4% thioglycollate broth (Sigma Aldrich, Germany). The tissue was homogenized through a strainer and filled up with 2mL (MLN, spleen), 3mL (lung), or 5mL (liver) of thioglycollate medium, respectively. In each case, 100uL tissue suspension of a 1:1000 dilution was plated either on McConkey agar plates for detection of gram negative bacteria (aerobic culture for 48h). Additionally, 100μl of 1:20000 diluted tissue homogenates were plated on Columbia blood agar plates and cultured under anaerobic or aerobic culture for 48h at 37°C. Colony forming units (CFU) were counted and calculated as CFU/g tissue.

### Antimicrobial peptide killing assay

Functional assay analysis to determine antimicrobial activity of antimicrobial peptides in biopsy extracts from mouse ileac lamina propria mucosae (LPM) was performed as follows and published previously [32]. Frozen tissue samples from manipulated and unmanipulated (CTL) mice were pulverized mechanically and diluted in 5% acetic acid supplemented with proteinase inhibitor cocktail (Sigma-Aldrich, Germany). Samples were vortexed and incubated on ice for 2h. After centrifugation (2000xg for 10min) supernatant was taken for cationic peptide sequence isolation while the pellet was taken for total protein extraction. The pellet was further incubated for 1h with 10M urea as well as 50mM dithiothreitol (DTT) at room temperature (RT) and at 95°C for another 5min. This was followed by lyophilization and reconstitution of the pellet by 0.01% acetic acid. For the isolation of the cationic fraction, Macro Preb CM Beads (Bio Rad, USA) were used and the supernatant incubated with the beads overnight on a plate shaker at 4°C. Subsequently the bead suspension was incubated with ammonium acetate for 10min on a shaker and the cationic fraction eluted with 20 μL of a 5% acetic acid. Lyophilization was also done as well as reconstitution of the eluate with 0.01% acetic acid. Protein concentration was analyzed by Bradford protein assay. For analysis *Escherichia (E*.*) coli* ATCC25922 and *Bacteroides (B*.*) vulgatus* ATCC8482 grown in Schaedler broth (BD Biosciences, Sparks, MD) were incubated at 37°C for 90 min with the total protein or cationic fraction extracted from 10 mg total biopsy protein in a final volume of 100 μL. The suspension was further incubated with membrane potential sensitive dye bis-(1,3-dibutylbarbituric acid) trimethine oxonol [32] (Invitrogen Life Technologies, Carlsbad, CA) was added in a concentration of 1 μg/mL to the suspension. After incubation at 37°C for 10 min, bacteria were pelleted, resuspended in PBS and the number of fluorescent bacteria analyzed by flow cytometry (FACS Calibur, BD Biosciences). Viable bacteria were distinguished from nonviable bacteria at single cell level. The antimicrobial activity was determined as percentage of depolarized bacteria compared with untreated controls.

### Quantitative RT-PCR

Total RNA of mucosal tissue samples were extracted by the use of Trizol (Life Technologies, Germany), according to the manufacturer’s instructions. cDNA was synthesized by the use of the High Capacity cDNA Reverse Transcription Kit (ThermoFisher Scientific, Germany). Expression of mRNA was quantified in triplicates by a real-time (RT)- PCR by SYBR Green QuantiTect Primer Assays (IL-1α, QT00113505; IL-1R1, QT00095256; Qiagen) or TaqMan probes (housekeeping gene GAPDH, NM_008084.2; IL-1β, Mm00434228; Thermo Fisher Scientific, Germany). Primers were designed for AMPs listed in **Table 1**. Quantitative PCR was performed with SYBR Green PCR Master Mix or TaqMan Gene Expression Master Mix (Thermo Fisher Scientific, Germany).

**Table 1:** Primers used for qPCR.

gene	primer	type	Primer sequence
Defa1	cryptdin 1	sense	CAGGCCGTATCTGTCTCCTT
		antisense	ATGACCCTTTCTGCAGGTTC
Defa4	cryptdin 4	sense	GTCCAGGCTGATCCTATCCA
		antisense	GGGGCAGCAGTACAAAAATC
mBD3	β-defensin 3	sense	GTTTGCATTTCTCCTGGTGC
		antisense	GCCTCCTTTCCTCAAACAACT
Lyz1	lysozyme 1	sense	GAGACCGAAGCACCGACTATG
		antisense	CGGTTTTGACATTGTGTTCGC
MUC2	mucin 2	sense	TGCCCAGAGAGTTTGGAGAGG
		antisense	CCTCACATGTGGTCTGGTTG
REG3G	REG3γ	sense	TTCCTGTCCTCCATGATCAAAA
		antisense	CATCCACCTCTGTTGGGTTCA

### Western blot

Jejunal and ileal mucosal tissues were lysed in 1x RIPA buffer and equal amounts were separated on 4–12% Bis-Tris NuPAGE gels in MES running buffer (Thermo Fisher Scientific, Germany). After wet blotting in 1x transfer buffer onto PVDF membranes (Merck, Darmstadt, Germany) proteins were blocked by 5% skim milk in Tris-buffered saline containing 0.1% Tween 20 and incubated with an anti–lysozyme-1 antibody (1:2000, abcam, Germany) and 1:2000 HRP-coupled secondary antibody (Cell Signaling Technologies, Germany). Proteins were detected by the Super Signal West Pico Plus Kit (Thermo Fisher Scientific, Germany). Afterwards the membranes were stripped with the Restore^TM^ Plus Western Blot stripping kit (Thermo Fisher Scientific, Germany) for 15min and incubated with an anti–beta actin antibody (1:25000, Sigma Aldrich, Germany) followed by a 1:200 diluted HRP-coupled secondary anti-mouse antibody (Cell Signaling, Germany).

### ELISA

Jejunal and ileal mucosal tissues were lysed in 1x RIPA buffer. Protein concentration of measured by Pierce^TM^ BCA Assay kit (ThermoFisher Scientific, Germany) and equal protein amounts (100 μg) were processed either by an IL-1α ELISA (R&D Systems, UK) or IL-1β ELISA (BD Biosciences, Germany) according to the manufactures instructions.

### Organoid cell culture

Organoids were generated from small bowel segments of WT and IL-1R1^-/-^ mice. Intestinal contents were first flushed out with cold PBS and then cut longitudinally with the intestinal lumen facing upward. After careful removal of villi using glass coverslips, the intestinal tissue was cut into small pieces, washed for 20 times in cold PBS and dissociated in 2mM EDTA at 4°C for 30 minutes. For plating the single crypts, around 100–200 crypts were suspended in 25 μL matrigel (BD Bioscience, Germany) per well in a 48-well plate. Crypts were cultivated in Advanced DMEM/F12 medium (Thermo Fisher Scientific, Germany) that was supplemented with 100ng/mL murine-Noggin, 50ng/mL murine endothelial growth factor (Thermo Fisher Scientific, Germany) and 500ng/mL R-Spondin.

### Agarose gel of qPCR-transcripts

PCR transcripts from gene expression analysis of the IL-1R1 in in vitro cultured organoids of WT and IL-1R1 deficient mice were applied on a 1.5% (w/v) agarose gel stained with ethidium bromide. A 100bp DNA-ladder was used as a marker. Strands of DNA were visualized by UV-absorbance as described by the manufacturer (Life Technologies, Germany).

### Immunohistochemical detection of mucin-2

Deparaffinized jejunal and ileal cross sections were cooked in pH 6.0 citrate buffer for 20 min, washed three times in PBS, incubated in 3% H_2_O_2_, washed again twice and permeabilized in 0.2% triton X-100, blocked in 5% goat serum and incubated with a rabbit anti-mouse anti-mucin-2 antibody (1:250, Santa Cruz Biotechnologies, Germany). Specimens were further processed with a Vectastain anti-rabbit kit (Vector laboratories, PK6101) following manufactures´ instructions. Antibody immunocomplexes were visualized by a common DAB staining. A counterstaining of the section finalized the procedure.

### Statistical analysis

Statistical analysis was performed with Prism V5.01 (GraphPad, San Diego, CA) using one-way or two-way analysis of variance followed by Bonferroni post-hoc test for group differences or Student’s t-test as indicated.

## Results

### Translocation and luminal overgrowth of bacteria during POI

**Fig 1:**
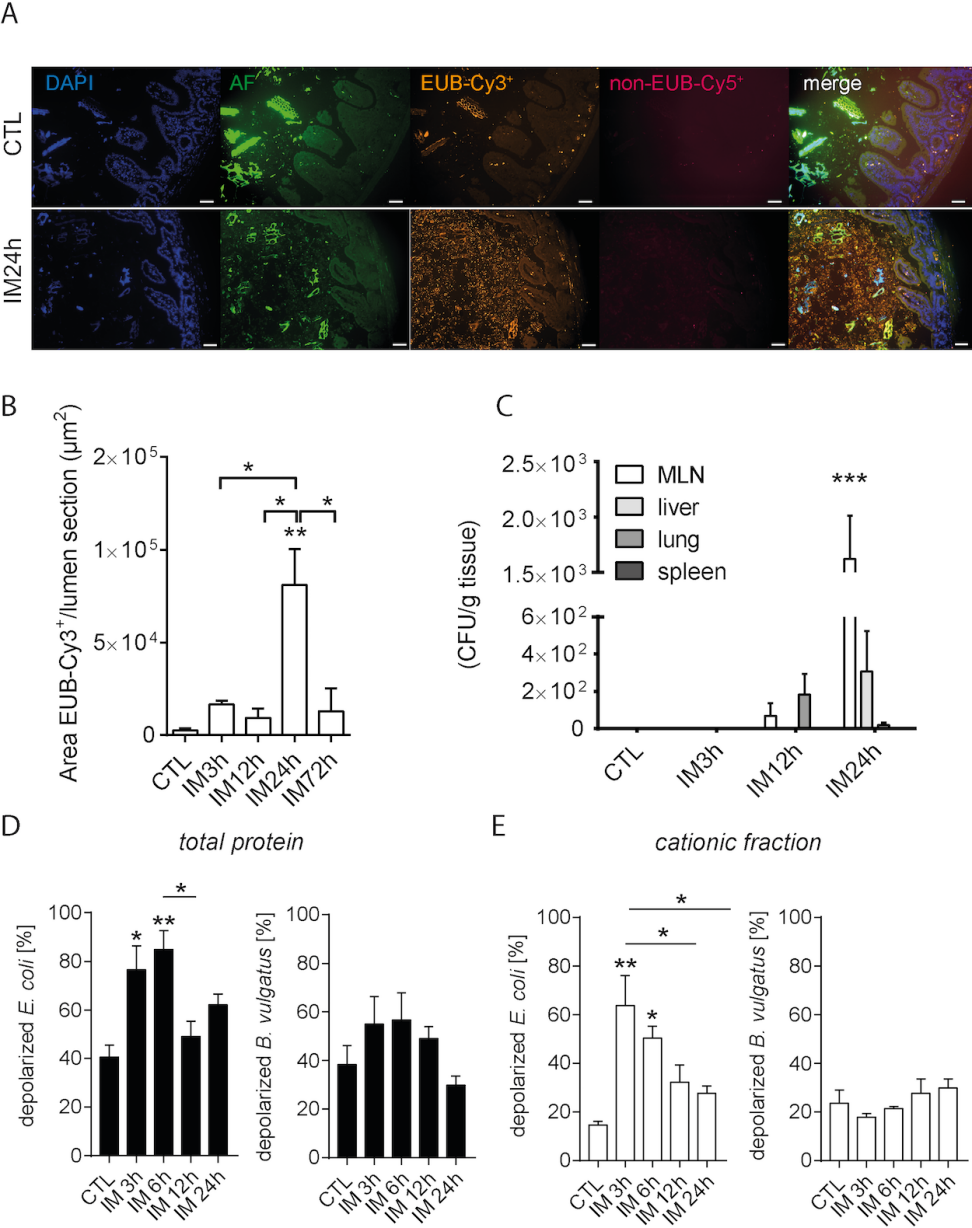
Translocation of luminal bacteria and increase of bacterial killing occurs during POI. (A) WT mice underwent IM. Representative photomicrographs of bacterial internalization into the post-operated gut mucosa (magnification ×200). Jejunal segments of intestinal manipulated mice and controls were subjected to FISH with universal bacterial DNA probes. DAPI-stained cell nuclei (blue) are shown for orientation. Cy2-staining shows intestinal auto-fluorescence (AF), whereby Cy3-labelled probe EUB338, complementary to the 16S rRNA in most microorganisms was combined with Cy5-labelled FISH probe NON-EUB338 to exclude unspecific probe binding. Photo images were obtained from at least three mice per group. (B) Bacteria (Cy3^+^) were analyzed in jejunal cross sections at different postoperative time points and in unmanipulated control mice. (C) Colony forming units (CFU) of MLN, liver, lung and spleen of manipulated WT mice were determined postoperatively and compared to WT naïve controls. (D+E) Flow cytometric analysis of the antimicrobial activity of the total protein [D] or the cationic fraction [E] of the mucosal tissue of intestinal manipulated mice against *E*. *coli* and *B*. *vulgatus*. Cell damage leads to an uptake of the membrane potential-sensitive dye DiBAC_4_ (3) in the bacteria and therefore to an increasing depolarization [%] compared to that of the untreated control. n = 5 for all groups. For all experiments, statistical analyses were performed with a 1-way ANOVA, followed by Bonferroni post hoc test. *p < 0.05, **p < 0.01, ***p < 0.001 vs. controls.

In our first experiment, we observed bacterial overgrowth in the upper GIT (jejunum) after induction of POI (IM24h) and further translocation of living bacteria from the lumen to the ME during the late phase of POI with the help of FISH technique (**Fig 1A**). Numbers of jejunal bacteria significantly increased 24h postoperatively (p<0.01) compared to controls and the 3h and 12h postoperative time points (p<0.05). Bacterial counts did not differ between the controls, 3h and 12h time points (**Fig 1B**). Translocation of living bacteria into MLN was observed 24h (p<0.001) but not 3h or 12h postoperatively on McConkey agar plates. The liver contained less bacteria and no bacterial translocation could be determined in the spleen and the lung (**Fig 1C**). At the earlier time point no significant bacterial translocation was detected. While McConkey agar selectively allows growth of gram negative bacteria we also plated the tissue homogenates on non-selective blood agar plates and cultured them under aerobic and anaerobic conditions for 48h. Under both conditions significant numbers of living bacteria in the mesenteric lymph nodes were observed 24h after IM. In the other organs bacteria could only be sporadically detected (**S1A and S1B Fig)**. Based on these findings, we were interested in the synthesis of AMPs by IECs and infiltrating immune cells as an important defense mechanism against microorganisms in the GIT. Therefore, we analyzed the depolarizing effect from AMPs isolated from ileal tissue after and without IM to two different bacterial strains, *E*. *coli* and *B*. *vulgatus* (**Fig 1D and 1E**). The incubation with *E*. *coli* demonstrated an increasing depolarization within the early phase of POI compared to unmanipulated controls, 3h (p<0.05) and 6h (p<0.01) postoperatively (**Fig 1D**). Effects returned to control levels 12h after IM and remained stable up to 24h postoperatively. Although the percentage of depolarization in the cationic fraction were below these of total protein, it demonstrated a similar pattern with an almost three times higher depolarization rate of *E*. *coli* 3h (p<0.01) and 6h (p<0.05) postoperatively in comparison to controls (**Fig 1E**). In contrast, *B*. *vulgatus* depolarization remained unaffected during POI (**Fig 1D and 1E**).

### Expression of antimicrobial proteins is differently regulated in the jejunal and ileal intestinum

**Fig 2:**
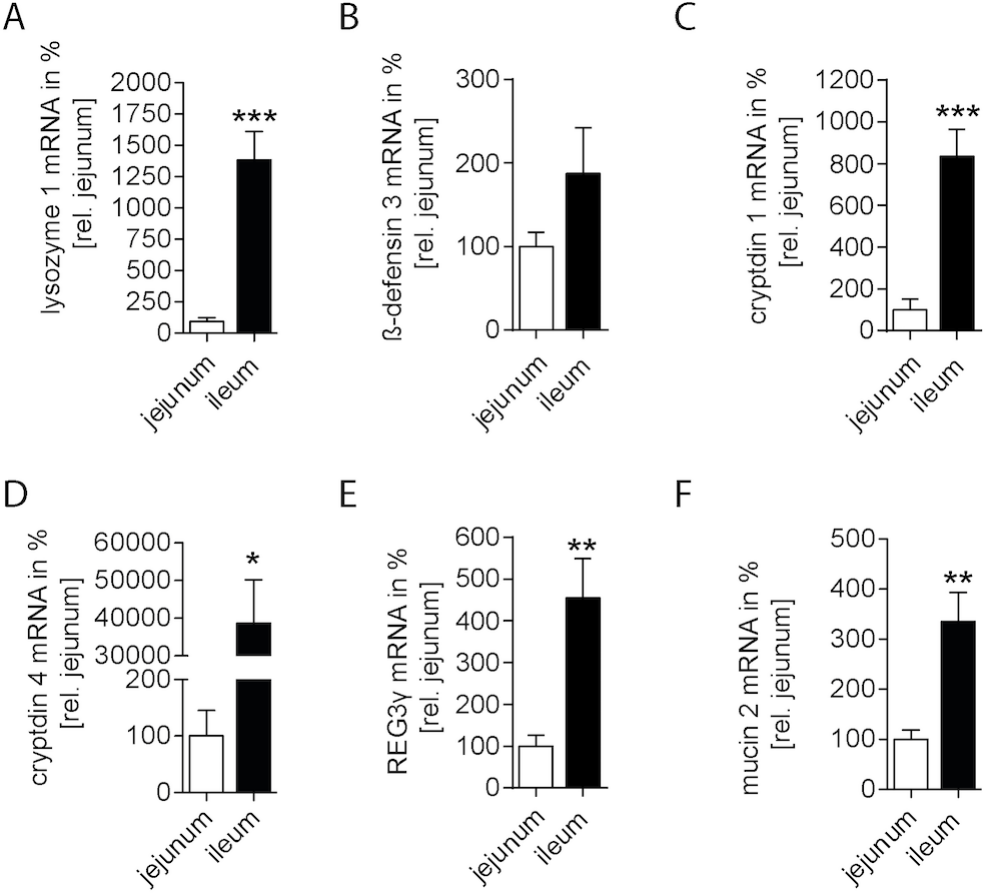
Basal antimicrobial proteins gene expression is differently regulated in the jejunum and ileum. Gene expression of antimicrobial proteins was quantified in ileal mucosa compared to jejunal mucosa of naïve control mice. (A) lysozyme 1; (B) β-defensin 3; (C) cryptdin 1; (D) cryptdin 4; (E) REG3γ; (F) mucin 2. n = 5 for all groups. Statistical analysis was done by unpaired Student’s t-test. *p < 0.05, **p < 0.01, ***p < 0.001 vs. jejunum.

**Fig 3:**
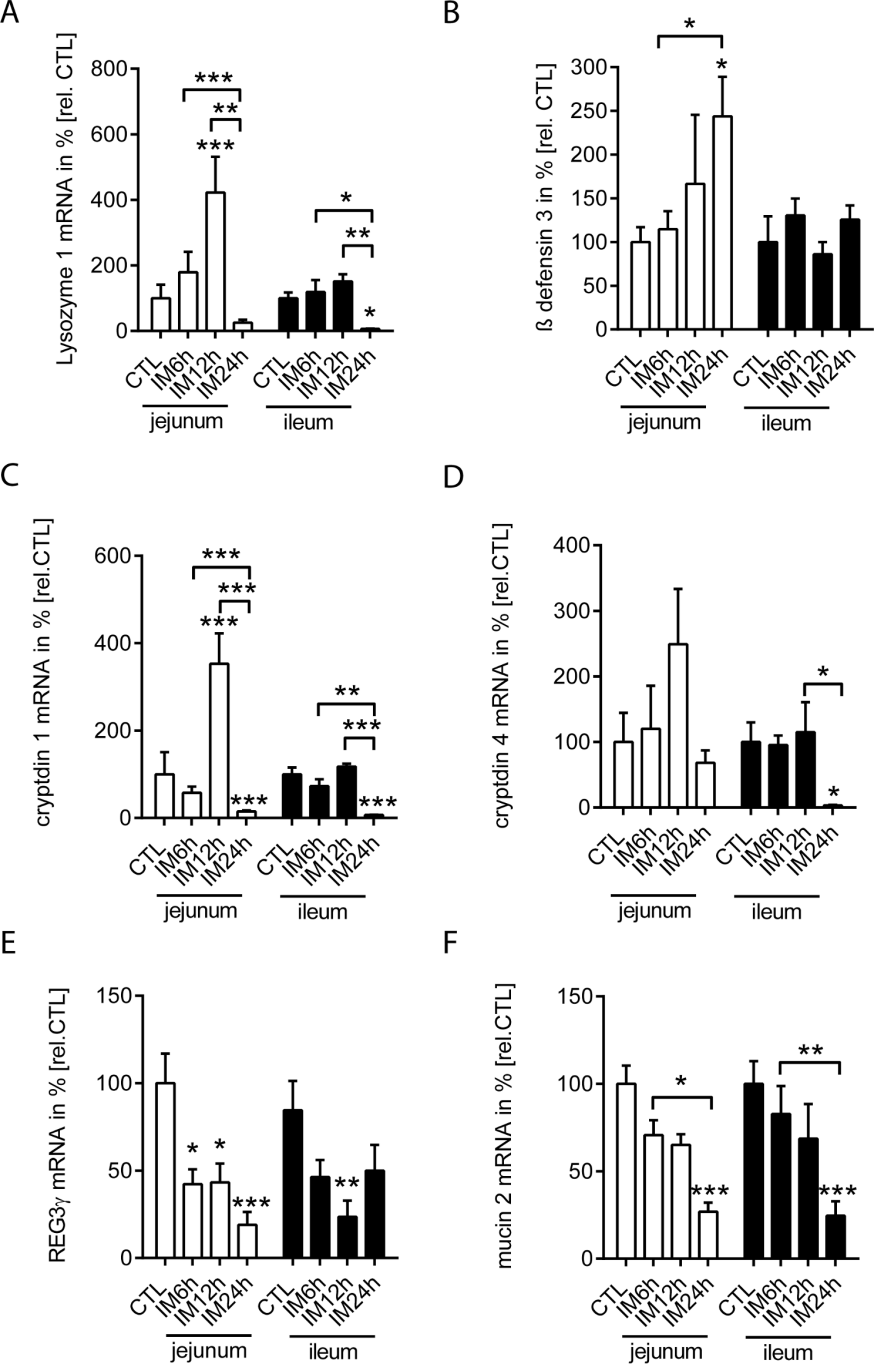
Postoperative gene expression of antimicrobial proteins is differently regulated in the jejunal and ileal intestinum. (A-F) WT mice underwent IM. Gene expression of lysozyme 1, β-defensin 3, cryptdin 1, cryptdin 4, REG3γ and mucin 2 was analyzed in mucosal tissue of jejunal and ileal bowel segments after indicated time-points and compared to naïve controls (CTL) or indicated time points. For comparison of transcript levels between the jejunum and ileum refer to S1 Fig. n = 5 for all groups. Statistical analysis was done by 2-way ANOVA, followed by Bonferroni post hoc test. *p < 0.05, **p < 0.01, ***p < 0.001 vs. controls or indicated time points.

Next we analyzed the gene expression of the following AMPs in the LPM of unmanipulated and intestinal manipulated WT mice: lysozyme 1, β-defensin 3, cryptdin 1 and 4, REG3γ as well as of mucin 2. Basal antimicrobial protein expression was compared between jejunal and ileal LPM samples (**Fig 2** and **S2 Fig**). In general, we observed a higher gene expression of lysozyme 1 (**Fig 2A**; p<0.001), cryptdin 1 (**Fig 2C**; p<0.001), cryptdin 4 (**Fig 2D**; p<0.05), REG3γ (**Fig 2E**; p<0.01) and mucin 2 (**Fig 2F**; p<0.01) in the ileum compared to the jejunum, while β-defensin 3 (**Fig 2B**) transcription levels did not differ between both bowel segments. In the time course of POI, we compared jejunual and ileal antimicrobial gene expression pattern after IM either to the non-manipulated segments (**Fig 3**) or between the jejunum and the ileum (**S2 Fig**). In the ileum, lysozyme 1 was downregulated 24h postoperatively (p<0.05) compared to unmanipulated controls and the 6h and 12h time point. In the jejunum, we observed a significant upregulation 6h after IM (**Fig 3A**). Ileal lysozyme 1 levels were higher in ileum 6h after IM (**S2A Fig)**. Immunoblot analyses confirmed the early increase of jejunal lysozyme expression but the protein remained upregulated also 24h after IM (**S3 Fig**). The ileal lysozyme 1 protein levels followed the non-significant trend observed at the transcription level and also dropped at IM24h. β-defensin 3 gene expression was only slightly upregulated during POI in the 24h postoperative jejunum (p<0.05), but not in the ileum (**Fig 3B**). Changes between the ileal and jejunal β-defensin 3 expression were hardly apparent 3h after IM (**S2B Fig**). Cryptdin 1 was upregulated at 12h in the jejunal segments (p<0.001), and downregulated at 24h (p<0.001, **Fig 3C**). In contrast, cryptdin 4 was only downregulated 24h postoperatively (and p<0.05) in the ileum. In both regions, the 3h postoperative time point was significantly higher in the ileum than the jejunum (**S2C and S2D Fig**). Postoperative jejunal REG-3γ gene expression was reduced at all postoperative time points. In the ileum, it was reduced only at 12h after IM (**Fig 3E**). The interregional comparison demonstrated elevated ileal Reg3γ levels 6h and 24h after IM compared to the jejunum (**S2E Fig**). Mucin 2 gene expression decreased in ileal and jejunal parts in a time dependent manner and reached lowest levels at 24h (p<0.01). An additional mucin 2 immunohistochemical staining (**S4 Fig**) identified a regular appearance of “mucin-2-filled” goblet cells within unmanipulated jejunal and ileal cross sections. After IM, mucin-2 stained goblet cells impose as “empty” cells that have significantly intracellular reduced loads of mucin-2. Of note, the postoperative expression did not differ between ileal and jejunal segments (Fig 3F and **S2F Fig**). Together, we identified a time dependent impact of IM on the mucosal antimicrobial gene expression whereby most genes were reduced in the late phase of POI.

### Mucosal IL-1R1 signaling plays a role in POI

**Fig 4:**
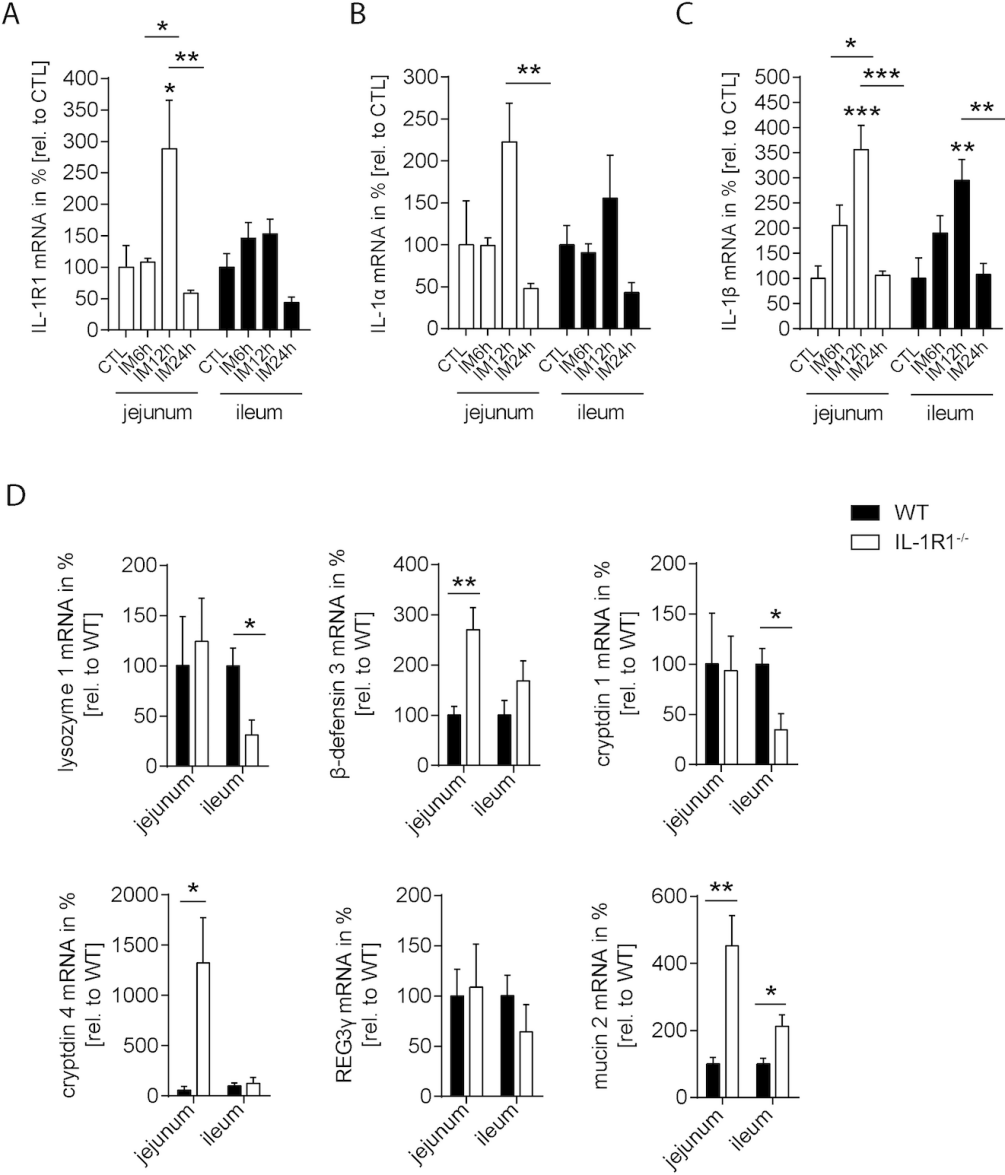
Mucosal IL-1R1 signaling is involved in POI. (A-C) WT mice underwent IM. Gene expression of (A) IL-1R1; (B) IL-1α and (C) IL-1β was analyzed in mucosal tissue of jejunal and ileal bowel segments after indicated time. (D) Basal gene expression of antimicrobial proteins was quantified in IL-1R1 deficient compared to control mice of jejunal and ileal mucosa. n = 5 for all groups. Statistical analysis was done by 1-way ANOVA, followed by Bonferroni post hoc test or t-test, respectively. *p < 0.05, **p < 0.01, ***p < 0.001 vs. indicated groups.

Based on the observed changes in mucosal defense mechanisms during POI, as well as the fact that the IL-1R1 plays a role in mucosal antimicrobial defense, we focused our subsequent analysis on IL-1 signaling. Previous work of our group has shown that IL-1R1 gene expression is not regulated in the muscularis externa within a 24h period following IM, while IL-1α and IL-1β were strongly induced [24]. In this study, we found that IL-1R1 gene expression was almost stable and only slightly upregulated 12h after POI onset in jejunal muscularis-free specimens (**Fig 4A**). IL-1α gene expression demonstrated a trend in upregulation 12h after surgery (**Fig 4B**). This increase turned out to be significantly on the protein level measured by ELISA (**S5A Fig**). In the ileum, no changes were observed, neither on RNA nor protein expression levels. In contrast, IL-1β RNA transcripts were upregulated in the jejunum and the ileum 12h postoperatively (jejunum p<0.001; ileum p<0.01) compared to CTL (**Fig 4C**). IL-1β protein levels however were not altered in the jejunum but reduced in the ileum 12h after IM (**S5B Fig**). Reasons for this discrepancy between RNA and protein measurement are discussed below. Intersegmental analysis between ileum and jejunum showed only 6h after IM a higher expression of IL-1R1 in the ileum compared to the jejunum (**S5C–S5E Fig**).

As IL-1R1 is also expressed in enterocytes as shown in intestinal epithelial crypt organoids (**S5F–S5H Fig**) we tested if IL-1R1 signaling is able to affect AMP expression in our POI model. Interestingly, AMP gene expression significantly differed in WT and IL-1R1 deficient mice under basal conditions (**Fig 4D**). Basal ileal lysozyme 1 and cryptdin 1 gene expression were lower in IL-1R1 deficient mice compared to WT mice, respectively. Contrarily, jejunal β-defensin 3, cryptdin 4 and mucin 2 as well as ileal mucin 2 gene expression were significantly higher in IL-1R1 deficient mice. Together these data indicate that IL-1R1 signaling affects mucosal antimicrobial gene expression in mice.

### IL-1R1 deficiency affects primarily jejunal AMP gene expression

**Fig 5:**
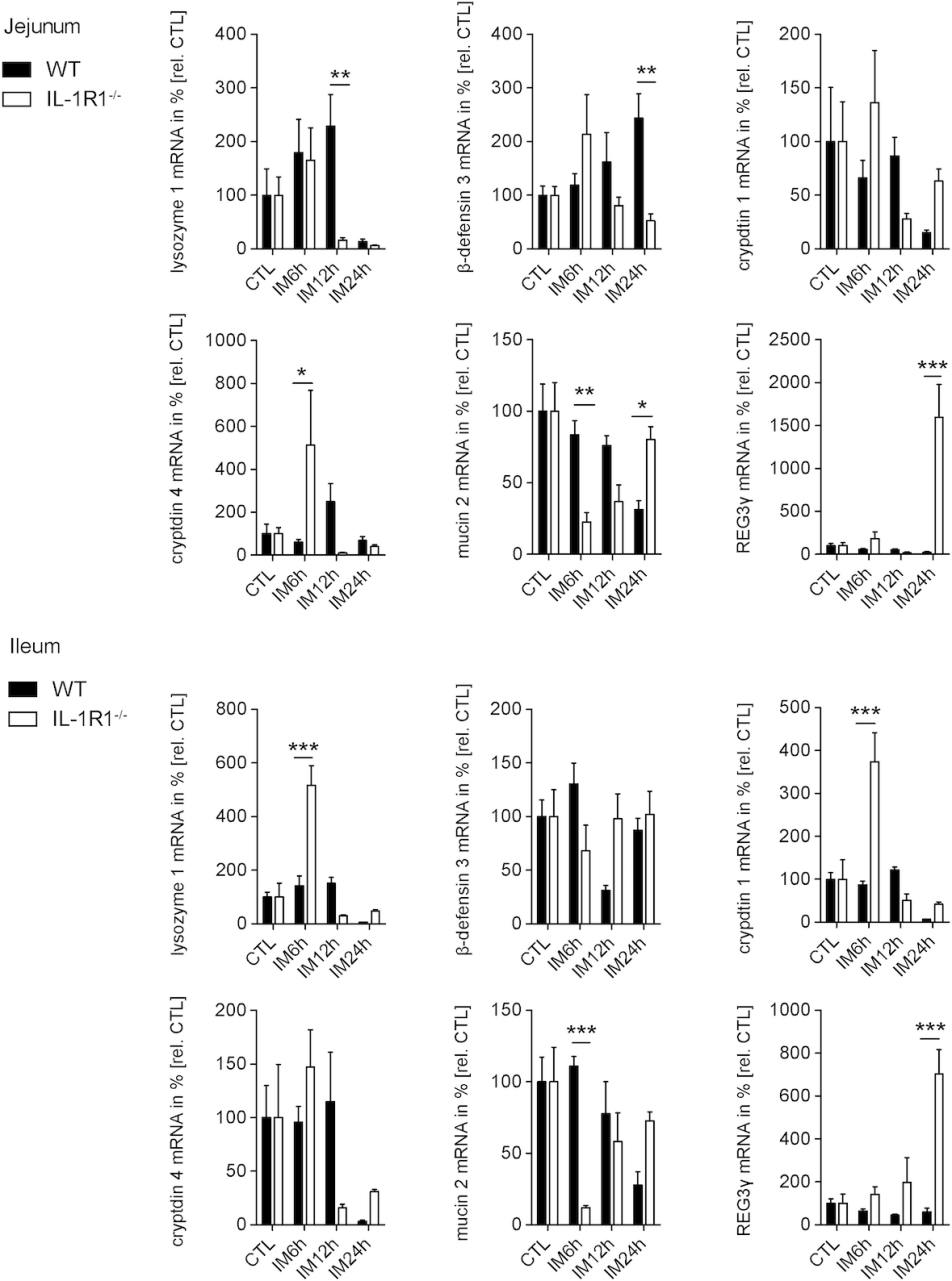
IL-1R1 deficiency primarily affects jejunal AMP gene expression. WT and IL-1R1 deficient mice underwent IM. Gene expression analysis of lysozyme 1, β-defensin 3, cryptdin 1 and 4, REG3γ as well as mucin 2 was performed in mucosal jejunum (upper half) and ileum (bottom half) after indicated time-points. n = 5 per group. Gene expression plotted as mean ± SEM. Statistical analysis was done by 2-way ANOVA, followed by Bonferroni post hoc test. *p < 0.05, **p < 0.01, ***p < 0.001 vs. CTL or WT.

Given by the basal difference in AMP gene expression between WT and IL1R1 mice we next compared the postoperative jejunal or ileal mucosal gene expression of IL-1R1^-/-^ with wildtype mice (**Fig 5**). In the jejunal mucosa, lysozyme 1 (p<0.001) or mucin 2 (p<0.01) were strongly reduced 12h or 6h after IM, respectively, in IL-1R1 deficient mice compared to WT mice **(Fig 5 Jejunum).** Mucin 2 levels recovered at 24h and were even increased compared to WT levels (p<0.05). β-defensin 3 (p<0.01) was also reduced but in the later phase of POI, 24h after IM. In contrast, cryptdin 4 showed a strong upregulation 6h postoperatively, while REG3γ was upregulated even more but during the late phase of POI (p<0.001).

While postoperative AMP transcription in the jejunum was strongly affected by IL-1R1 signaling, ileal AMP expression was changed to a lesser extent (**Fig 5 Ileum**). Mucin2 showed a similar reduction 6h after IM under IL-1R1 deficiency, whereas lysozyme 1 and cryptdin 1 transcripts were increased at this time point. β-defensin 3 and cryptdin 4 were not altered, whereas REG3γ (p<0.001 at 24h) demonstrated also a strong increase in IL-1R1 mice.

Together our results show that POI development is associated with bacterial overgrowth in the upper part of the GIT and higher depolarization activity in the mucosa of manipulated mice compared to unmanipulated controls. This is accompanied by time-dependent changes in AMP expression during POI that primarily affects the jejunal and ileal mucosa of WT mice during late phase of POI. This regulation partially depends on IL-1R1 signaling indicating that IL-1 signaling is not only involved in the inflammatory muscularis externa response but also in the antimicrobial mucosal defense during POI.

## Discussion

Previous work from our group and others have already shown that POI is associated with a strong inflammation of the bowel wall, particularly of the ME [33, 34], which includes production of a variety of cytokines and chemokines that trigger infiltration of circulating leukocytes into the bowel wall. Interestingly, the inflammatory response is much less pronounced in the mucosa with a dramatically lower production of cytokines and chemokines after surgery [5]. On the other hand, previous studies in rodents [5] and humans [8, 35] indicated that surgical manipulation of the bowel can lead to translocation of luminal bacteria and further dissemination to distinct organs via abdominal lymphatics or the bloodstream. This demonstrated that the barrier integrity of the mucosa is disturbed during POI. Herein we show that translocation occurs in the late phase of POI (24h postoperatively) but not in the early phase (3h) and affects aerobic and anaerobic bacteria. The translocated living bacteria are able to pass the bowel wall and drain to the liver and the mesenteric lymph nodes but not into the lung or spleen. This indicates that the mesenteric lymph nodes are able to retain translocating luminal bacteria to prevent systemic dissemination. The lymphatic route is described as a second line of host defense to reduce further translocation to peripheral organs [36]. Bacterial translocation is characteristic for luminal bacterial overgrowth and was already observed in oral antibiotic therapy, endotoxic shock, starvation, parenteral nutrition, bowel obstruction, etc. [37]. When the intestinal mucosa becomes damaged, like in endotoxemia shock, thermal injury or hemorrhagic shock, bacteria are even able to reach the liver via the portal vein [36].

Since translocation of bacteria to peripheral organs as the liver requires a tight contact to the epithelium which produces AMPs and enzymes with antimicrobial activity to limit the epithelial attachment, we hypothesized that surgical manipulation may affect the epithelial antimicrobial activity. As many AMPs are known to destabilize or to disrupt the cytoplasmic membrane of bacteria [38] we determined the antimicrobial activity during POI by analyzing the depolarizing effect of mucosal tissue extracts [32] against two bacterial specimen that are commonly present in the intestinal flora, the Gram-negative facultative anaerobe species *E*. *coli* and the anaerobic species *B*. *vulgatus* [39]. Total protein as well as cationic extracts from wildtype bowel biopsies showed a high bactericidal effect against *E*. *coli* during early phases of POI development, whereas the activity against *B*. *vulgatus* remained unaffected up to 24h after surgery. This indicates that in response to surgery, selective AMPs may be produced or released from secretory granules with a greater specificity for killing *E*. *coli* than *B*. *vulgatus*. Unfortunately, the wide range of AMPs and the lack of deficiency or blocking strategies make it difficult to further identify single mucosal peptides with this method. Additionally, surgery induced antimicrobial activity is upregulated predominantly in the early phase of POI what may explain the absence of living translocated bacteria in the liver and mesenteric lymph nodes in this phase. Given that the antimicrobial activity is postoperatively modulated we next analyzed the generation of different AMPs in the upper and lower parts along the small bowel. Almost all tested AMPs demonstrated higher gene expression levels in the ileum than in the jejunum under basal conditions. This can be explained by the in general higher load of bacteria in the ileum which is around 10^5^-fold higher in the distal small bowel compared to the proximal parts [40]. In comparison to these basal conditions, ileal AMP gene expression undergoes important changes during POI, particularly in the late phase where most tested enzymes and AMPs, except β-defensin 3 and REG3γ, were downregulated. Although gene expression analyses are still the gold standard in AMP detection, at least for the detection of defensins and REG3γ, we were able to detect protein level changes for mucin 2 and lysozyme 1. In general, both followed the trends observed in mRNA levels. Furthermore, the decreased gene expression of most molecules, touching bottom levels 24h after IM, is in line with the increased bacterial translocation to mesenteric lymph and with the functional decrease of intestinal motility in the late phase of POI. Subsumed, our results indicate an important role of AMPs in regulation of the luminal microbial load while protecting the intestinal barrier [13, 41, 42].

AMP secretion in the lung and skin was shown to be regulated via the IL-1R1 ligands IL-1α and IL-1β [17–19]. Although less is known about the involvement of IL-1R1 signaling in intestinal AMP expression, IL-1β was recently shown to enhance human β-defensin 2 and mucin 2 production in colonic epithelial cells [43]. Herein we observed that mucosal IL-1R1 gene expression is almost stable over a time course of 24h after surgery as it is in the postoperative muscularis [24]. The only increase was observed in the jejunal mucosa 12h after IM where IL-1α mRNA and protein levels were also increased. Of note, IL-1β gene expression was more prominently upregulated than IL-1α in both, the jejunal and ileal mucosa. However, this increase was not obvious on the protein level. This may be due to the fact that IL-1β detection in tissue specimen is not trivial because IL-1β in contrast to IL-1α, undergoes inflammasome-mediated maturation during inflammation and this maturation is not detectable by common ELISAs in tissue specimen. Therefore, IL-1β protein measurement is of limited informative values and only strong expression changes are likely to be detected by tissue ELISA.

Given that IL-1 ligand expression is altered in the postoperative mucosa, we questioned if IL-1R1 signaling may affect AMP production during POI. As intestinal epithelial cells are the major source of AMPs we investigated IL-1R1 expression in intestinal epithelial organoid cultures which provide a pure epithelial culture system containing all kinds of differentiated epithelial cells [44]. These organoids expressed the IL-1R1 and the specificity of specimen was demonstrated by absence of transcripts in IL-1R1^-/-^ organoids. This indicates that epithelial cells in principal are able to detect and respond to IL-1R1 ligands. Indeed, we overserved substantial changes in the AMP gene expression profile, particularly in the upper GI- tract in mice lacking the IL-1R1. IL-1R1 deficiency resulted in a strong reduction of lysozyme 1, β-defensin 3 and cryptdin 4. Contrarily, mucosal REG3γ, the mouse orthologue for human REG3α [45], was massively increased in late phase POI in the upper and lower GI-tract. REG3γ belongs to C-type lectins and is effective against gram-positive bacteria via binding to peptidoglycan [46]. It is essential for maintaining a 50μm zone that physically separates luminal bacteria from the small bowel epithelial surface [14]. As IL-1R1 also signals via MyD88 signaling the latter may be partially reduced in IL-1R1 deficient mice. The strong REG3γ upregulation may be a counter regulatory mechanism triggered by other MyD88 stimulating routes, including TLR signaling. Of note, C-type lectins including REG3γ were shown to be activated by TLR/MyD88 signaling in the intestine [13, 45]. Supporting, TLR expression in epithelial cells, particularly paneth cells, tightly regulates AMP production in a TLR/MyD88 dependent manner and cryptdins as well as REG3γ were reduced in MyD88 deficient mice [47, 48].

In conclusion, small bowel AMP production is reduced in late phase POI and what in turn may facilitate translocation of luminal bacteria across the epithelium. These changes predominantly affect the upper GI-tract which is sparely colonialized by bacteria under healthy conditions but suffers from bacterial overgrowth during POI. Furthermore, IL-1R1 is involved in AMP production during POI. This new mucosal function supplements our knowledge about further IL-1R1 signaling contribution to the pathophysiology of POI.

## Supporting information

S1 Fig Differentiation of translocated luminal bacteria in aerobe and anaerobe during POI. WT mice underwent IM. Colony forming units (CFU) of (A) aerobic or (B) anaerobic bacteria in MLN, liver, lung and spleen of manipulated WT mice were determined postoperatively and compared to WT naïve controls. n = 5 for all groups. For all experiments, statistical analyses were performed with a 1-way ANOVA, followed by Bonferroni post hoc test. *p < 0.05, **p < 0.01 vs. controls.
(TIF)

S2 Fig Antimicrobial proteins gene expression is differently regulated in the jejunum and ileum. Gene expression of antimicrobial proteins was quantified between the ileal and jejunal mucosa of naïve control mice and mice that underwent IM (IM6h, IM12h, IM24h). (A) lysozyme 1; (B) β-defensin 3; (C) cryptdin 1; (D) cryptdin 4; (E) REG3γ; (F) mucin 2. n = 5 for all groups. Statistical analysis was done by 2-way ANOVA, followed by Bonferroni post hoc test. *p < 0.05, **p < 0.01, ***p < 0.001 vs. jejunum.
(TIF)

S3 Fig Lysozyme 1 protein expression in jejunal and ileal mucosa during POI. Protein expression of Lysozyme 1 and ß-actin were analyzed in jejunal and ileal mucosa of naïve control mice and IM mice (IM6h, IM12h, IM24h) by western blot analysis.
(TIF)

S4 Fig Effect of POI on Mucin 2 expression in small intestinal goblet cells. Mucin 2 protein expression was analyzed in jejunal and ileal cross sections of naïve and IM mice (IM6h, IM12h, IM24h, n = 3 per group). Representatives pictures images were taken by microscopy with at a 200× magnification.
(TIF)

S5 Fig Mucosal IL-1R1 ligand regulation during POI and its expression profile in crypt organoids. (A-B) Protein expression of IL-1α and IL-1β was analyzed in jejunal and ileal mucosa of naïve mice and 12h postoperatively by ELISA. n = 5 for all groups. Statistical analysis was done by Student’s t-test. *p < 0.05 vs. control. (C-E) Gene expression of antimicrobial proteins was quantified between the ileal and jejunal mucosa of naïve control mice and mice that underwent IM (IM6h, IM12h, IM24h), (C) IL-1R1; (D) IL-1α; (E) IL-1β. n = 5 for all groups. Statistical analysis was done by 2-way ANOVA, followed by Bonferroni post hoc test. *p < 0.05, **p < 0.01, ***p < 0.001 vs. jejunum. To confirm IL-1R1 expression in IECs, we cultivated isolated murine crypts from small intestine of WT and IL-1R1^-/-^ mice and cultured them as crypt organoid cultures. (F) IECs grew as epithelial organoids and were harvested 5 days after plating. Images are representative of three mice per group. Pictures were taken at a 100× magnification. (G) Quantitative gene expression and (H) common PCR analysis showed that IECs from the small intestine of WT mice express the IL-1R1 under basal conditions. IL-1R1 transcripts were absent in organoid cultures of IL-1R1 deficient mice.
(TIF)
