# Supplementary figures and images for: Intestinal manipulation affects mucosal antimicrobial defense in a mouse model of postoperative ileus
Source: PLoS One. 2018 Apr 13;13(4):e0195516. doi: 10.1371/journal.pone.0195516 (PMC5898729; doi:10.1371/journal.pone.0195516)

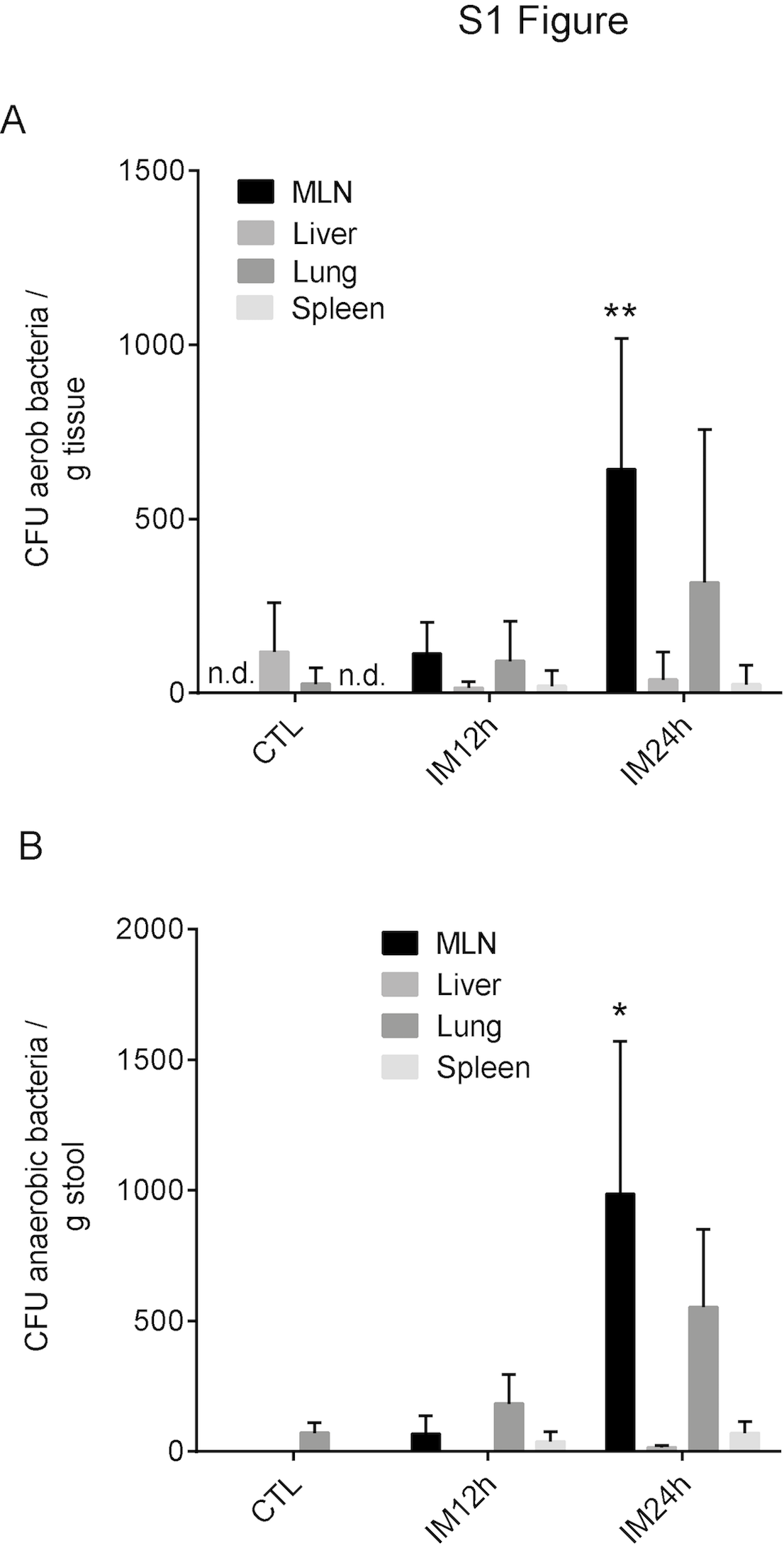

Supplement: S1 Fig — WT mice underwent IM. Colony forming units (CFU) of (A) aerobic or (B) anaerobic bacteria in MLN, liver, lung and spleen of manipulated WT mice were determined postoperatively and compared to WT naïve controls. n = 5 for all groups. For all experiments, statistical analyses were performed with a 1-way ANOVA, followed by Bonferroni post hoc test. *p < 0.05, **p < 0.01 vs. controls. (TIF) [file pone.0195516.s001.tif]

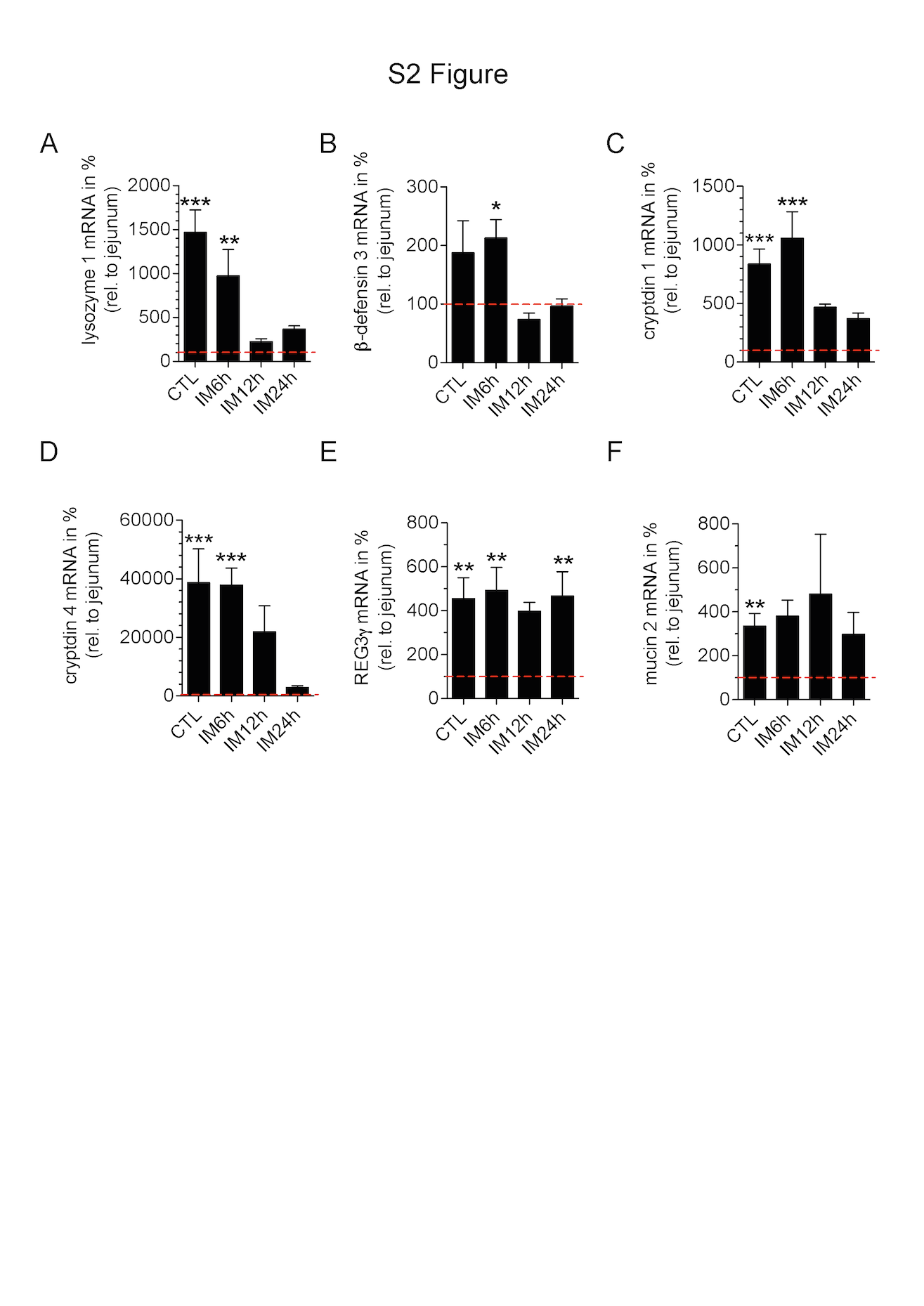

Supplement: S2 Fig — Gene expression of antimicrobial proteins was quantified between the ileal and jejunal mucosa of naïve control mice and mice that underwent IM (IM6h, IM12h, IM24h). (A) lysozyme 1; (B) β-defensin 3; (C) cryptdin 1; (D) cryptdin 4; (E) REG3γ; (F) mucin 2. n = 5 for all groups. Statistical analysis was done by 2-way ANOVA, followed by Bonferroni post hoc test. *p < 0.05, **p < 0.01, ***p < 0.001 vs. jejunum. (TIF) [file pone.0195516.s002.tif]

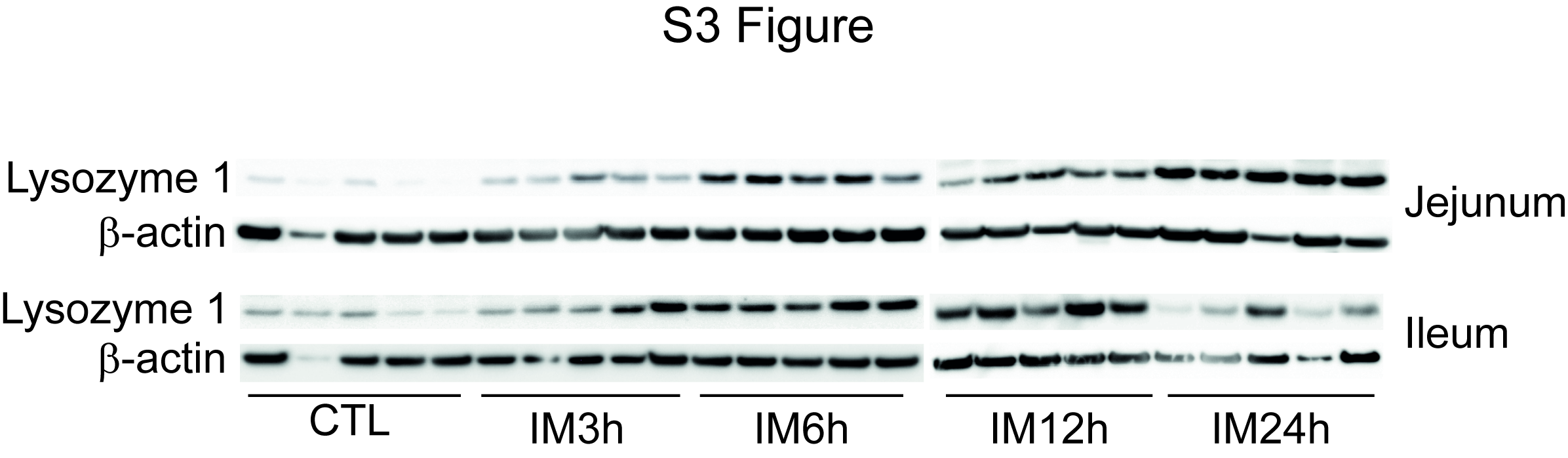

Supplement: S3 Fig — Protein expression of Lysozyme 1 and ß-actin were analyzed in jejunal and ileal mucosa of naïve control mice and IM mice (IM6h, IM12h, IM24h) by western blot analysis. (TIF) [file pone.0195516.s003.tif]

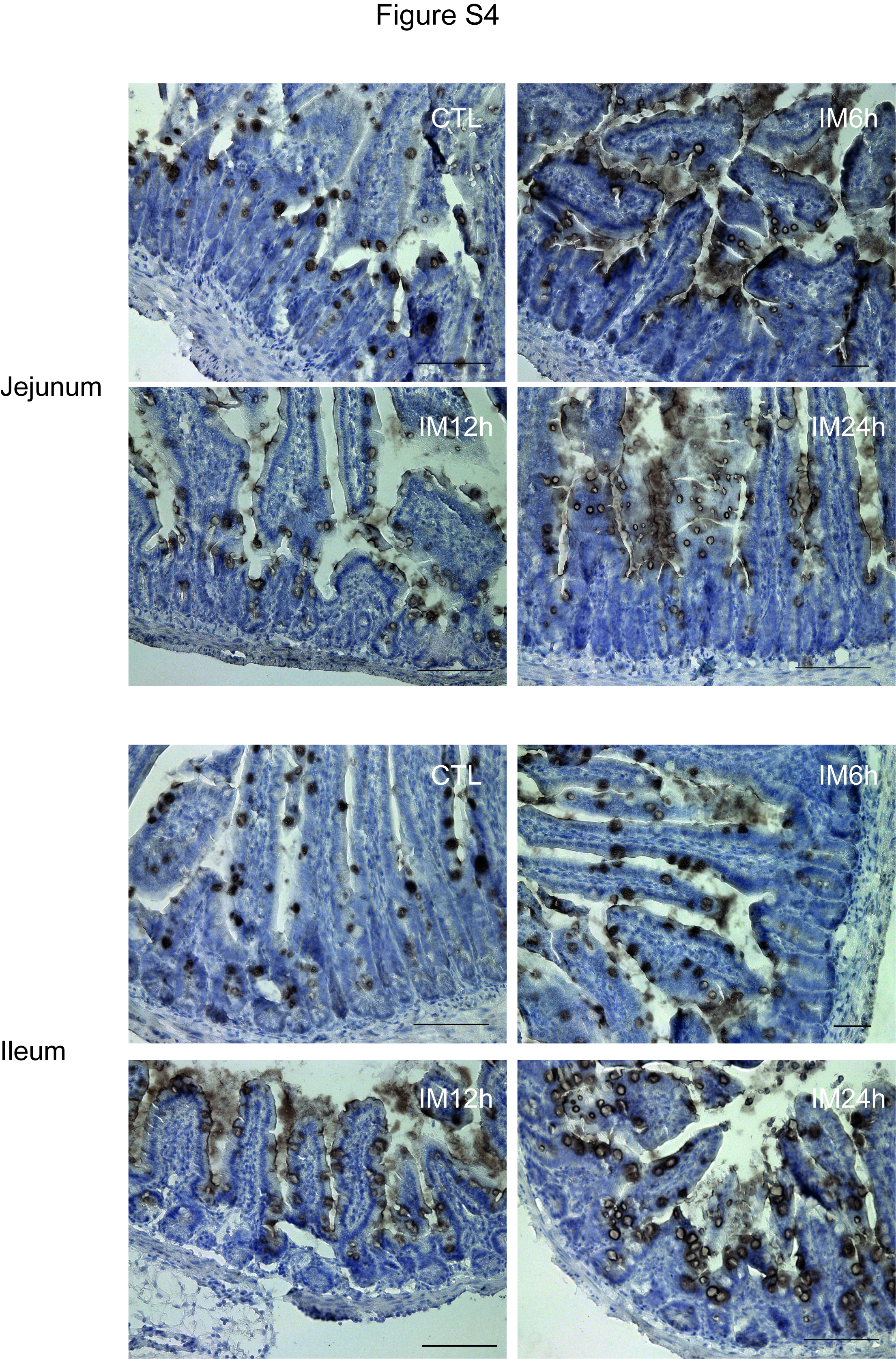

Supplement: S4 Fig — Mucin 2 protein expression was analyzed in jejunal and ileal cross sections of naïve and IM mice (IM6h, IM12h, IM24h, n = 3 per group). Representatives pictures images were taken by microscopy with at a 200× magnification. (TIF) [file pone.0195516.s004.tif]

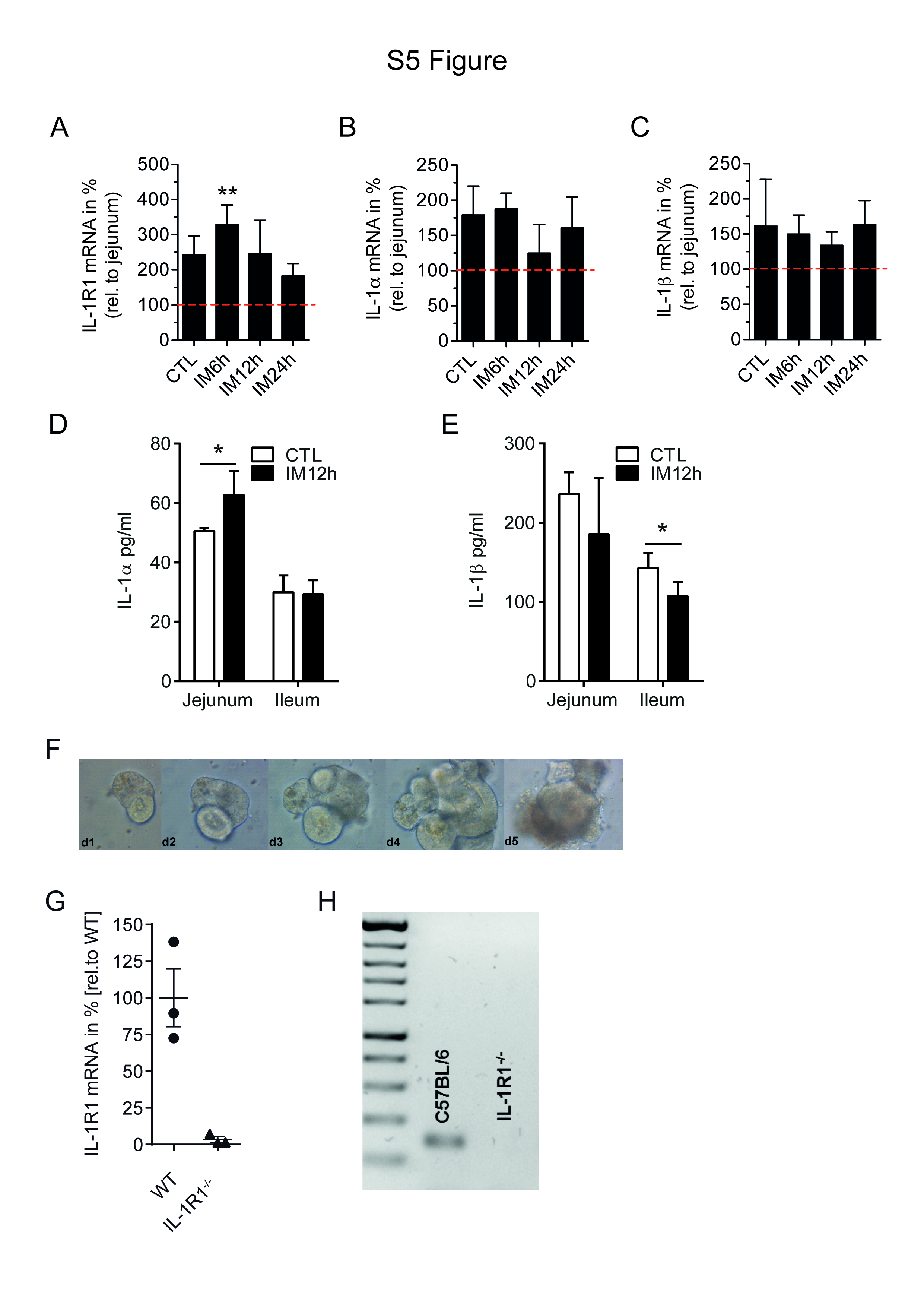

Supplement: S5 Fig — (A-B) Protein expression of IL-1α and IL-1β was analyzed in jejunal and ileal mucosa of naïve mice and 12h postoperatively by ELISA. n = 5 for all groups. Statistical analysis was done by Student’s t-test. *p < 0.05 vs. control. (C-E) Gene expression of antimicrobial proteins was quantified between the ileal and jejunal mucosa of naïve control mice and mice that underwent IM (IM6h, IM12h, IM24h), (C) IL-1R1; (D) IL-1α; (E) IL-1β. n = 5 for all groups. Statistical analysis was done by 2-way ANOVA, followed by Bonferroni post hoc test. *p < 0.05, **p < 0.01, ***p < 0.001 vs. jejunum. To confirm IL-1R1 expression in IECs, we cultivated isolated murine crypts from small intestine of WT and IL-1R1-/- mice and cultured them as crypt organoid cultures. (F) IECs grew as epithelial organoids and were harvested 5 days after plating. Images are representative of three mice per group. Pictures were taken at a 100× magnification. (G) Quantitative gene expression and (H) common PCR analysis showed that IECs from the small intestine of WT mice express the IL-1R1 under basal conditions. IL-1R1 transcripts were absent in organoid cultures of IL-1R1 deficient mice. (TIF) [file pone.0195516.s005.tif]
